# Improved Serodiagnostic Performance for Lyme Disease by Use of Two Recombinant Proteins in Enzyme-Linked Immunosorbent Assay Compared to Standardized Two-Tier Testing

**DOI:** 10.1128/JCM.01004-17

**Published:** 2017-09-25

**Authors:** Gary L. Bradshaw, R. Kelley Thueson, Todd J. Uriona

**Affiliations:** Ross Southern Laboratories, Spanish Fork, Utah, USA; Memorial Sloan Kettering Cancer Center

**Keywords:** 2-tier testing, Lyme disease

## Abstract

The most reliable test method for the serological confirmation of Lyme disease (LD) is a 2-tier method recommended by the CDC in 1995. The first-tier test is a low-specificity enzyme-linked immunosorbent assay (ELISA), and the second-tier tests are higher-specificity IgG and IgM Western blots. This study describes the selection of two Borrelia burgdorferi recombinant proteins and evaluation of their performance in a simple 1-tier test for the serological confirmation of LD. These two proteins were generated from (i) the full-length *dbpA* gene combined with the invariable region 6 of the *vlsE* gene (DbpA/C6) and (b) the full-length *ospC* gene (OspC). The expressed DbpA/C6 and OspC proteins were useful in detecting anti-Borrelia IgG and IgM antibodies, respectively. A blind study was conducted on a well-characterized panel of 279 human sera from the CDC, comparing ELISAs using these two recombinant antigens with the 2-tier test method. The two methods (DbpA/C6-OspC versus 2-tier test) were equivalent in identifying sera from negative-control subjects (99% and 100% specificity, respectively) and in detecting stage II and III LD patient sera (100% and 100% sensitivity). However, the DbpA/C6-OspC ELISA was markedly better (80% versus 63%) than the 2-tier test method in detecting anti-Borrelia antibodies in stage I LD patients. The findings suggest that these antigens could be used in a simple 1-tier ELISA that is faster to perform, easier to interpret, and less expensive than the 2-tier test method and which is better at detecting Borrelia-specific antibodies in sera from patients with stage I LD.

## INTRODUCTION

Lyme disease (LD) continues to be the most common vector-borne disease in North America (1), with increasing incidence in the United States and Canada (2). The primary agent of LD in North America is the spirochete Borrelia burgdorferi sensu stricto, which is transmitted chiefly by the tick vectors Ixodes scapularis and Ixodes pacificus (3). The diagnosis of LD is based on patient history, clinical presentation, and serology (1, 3). The observation of erythema migrans (EM), which is present in 70 to 80% of early LD patients (1, 3), is very important in the detection and diagnosis of this stage of disease and is considered pathognomonic in areas of endemicity (4). Direct detection of the spirochete, either by culture or by PCR amplification of Borrelia genes, is often unreliable due to the small number of organisms present in any sample or stage of infection (2, 3). Therefore, serology remains the most important confirmatory step in the diagnosis of LD (5). As with many infectious diseases, early detection and confirmation of LD are difficult to establish, but they are crucial in the management of the disease. Early treatment for LD can mitigate or even prevent the complications of late-stage illness, which can adversely affect the heart, nervous system, and joints and can persist for months or even years (6).

The most reliable serological testing method for the diagnosis of LD, which was recommended by the Centers for Disease Control and Prevention (CDC) in 1995 (7), continues to be a 2-tier test method. The first tier is a screening assay and is most commonly an enzyme-linked immunosorbent assay (ELISA), using as the antigens either a whole-cell lysate of B. burgdorferi or specific recombinant proteins or peptides (8). It is typically of low specificity but high sensitivity. If the first-tier test is negative, the sample is considered to be negative, and no further testing is advised. However, if the first-tier test is positive or equivocal, it is recommended that second-tier testing be done. The second-tier tests are Western blot (WB) assays for both IgG and IgM antibodies. Although they also use whole-cell lysates of B. burgdorferi or specific recombinant proteins as antigens, they provide higher specificity than the first-tier test due to the algorithms used for interpretation. For LD IgM WBs to be considered positive, at least 2 of 3 bands (p23, p39, or p41) must be determined to be positive, and for LD IgG WBs to be considered positive, at least 5 of 10 bands (p18, p23, p28, p30, p39, p41, p45, p58, p66, or p93) must be read as positive (5, 7).

WB assays are considered technically complex to perform and are, most often, subjective in their interpretation (5, 9). In contrast, ELISAs are relatively straightforward to perform, can be quantitative, and are nonsubjective in their interpretation. The purpose of this study was to develop one or two recombinant antigens from B. burgdorferi which could be used in the development of a 1-tier ELISA for the confirmation and diagnosis of LD and which could yield overall specificities and sensitivities equal to or better than those of the 2-tier method for the detection of Borrelia-specific antibodies in human sera.

## RESULTS

### Gene expression and reactivity of proteins.

**FIG 1:**
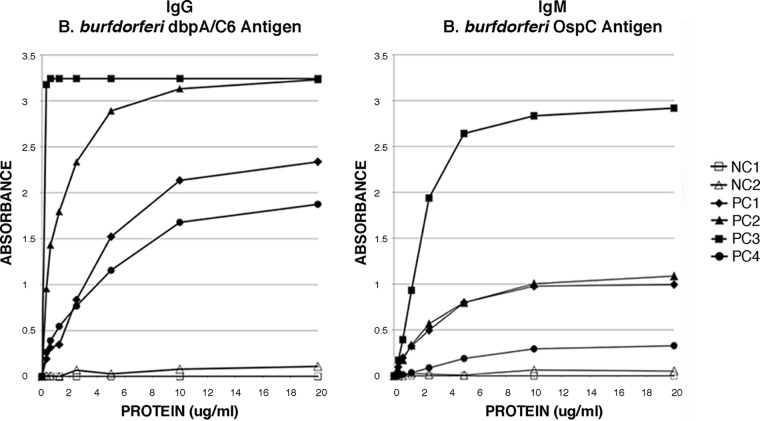
Antigen titrations of DbpA/C6 for IgG detection and of OspC for IgM detection in an ELISA format. NC, negative control; PC, positive control. The optimal coating concentration was determined to be ∼10 μg/ml, as this was the minimal tested value giving the greatest discrimination between the positive- and negative-control sera.

As part of the initial phase of this study, a number of B. burgdorferi proteins considered to be important in the serology of LD were generated in our laboratory as recombinant proteins expressed in Escherichia coli; these included DbpA, OspC, p28, p30, p39, p41, p45, p58, p66, p93, and VlsE. Also included in the studies was the synthetic peptide C6 as well as a recombinant fusion protein which was comprised of both DbpA and C6 (DbpA/C6). Each of these purified antigens was titrated in an ELISA format to demonstrate their specific activity and to determine their optimal coating concentrations. Titration curves for the antigens DbpA/C6 for the detection of IgG antibodies and OspC for the detection of IgM antibodies are shown as examples in Fig. 1. The optimal coating concentration is defined here as the minimum concentration of antigen yielding maximum discrimination between the positive- and negative-control sera. For both DbpA/C6 and OspC, the optimal coating concentration was determined to be approximately 10 μg/ml (Fig. 1).

These recombinant proteins varied substantially in their reactivity with Borrelia-specific IgG and IgM antibodies when tested against the LD positive and negative-control sera from MarDx. The DbpA, VlsE, and DbpA/C6 antigens each showed a high degree of reactivity with IgG anti-Borrelia antibodies, while OspC, p39, and p41 were very reactive with both IgG and IgM anti-Borrelia antibodies. However, it was observed that p39, p41, and VlsE each also showed a significant degree of reactivity with some of the negative-control sera as well (results not shown). Based on these results, three recombinant proteins (DbpA, DbpA/C6, and OspC) were chosen for further evaluation. To directly compare the reactivities of these three proteins, along with the synthetic C6 peptide, a panel of 18 LD positive-control sera from MarDx was used. Cutoff and equivocal values were determined using a panel of sera from LD-negative subjects accumulated to that point in the study, which were included in each assay. As shown, in these comparison tests, only DbpA/C6 identified all of the LD control sera as positive for IgG (Table 1), and only OspC identified all as positive for IgM (Table 2). Also of note, as shown in Table 1, is that the fusion protein DbpA/C6 elicited an IgG reaction in more than half the sera tested that was stronger than the combined results of both of its component parts. Based on these and other similar results, the field of recombinant proteins was narrowed to only two, DbpA/C6 (for the detection of IgG) and OspC (for the detection of IgM), for more extensive evaluation in blind studies using well-characterized LD serum panels received from the CDC (Fort Collins, CO).

**TABLE 1:** Comparisons of the DbpA/C6, DbpA, C6, and OspC antigens for their ability to detect Borrelia-specific IgG antibodies in LD-positive patient sera

Serum^*a*^	Absorbance value and result^*b*^			
	DbpA/C6	DbpA	C6	OspC
L1	2.757 P	0.761 P	0.445 N	3.236 P
L2	3.333 P	1.800 P	2.056 P	3.432 P
L3	3.190 P	1.426 P	3.173 P	3.403 P
L4	3.192 P	2.783 P	1.501 P	1.181 N
L5	1.341 P	0.186 N	0.087 N	3.068 P
L6	3.359 P	2.842 P	0.716 E	2.942 P
L7	3.146 P	2.878 P	0.418 N	1.095 N
L8	3.379 P	3.379 P	0.087 N	3.344 P
L9	2.672 P	0.739 P	0.716 E	3.166 P
L10	2.345 P	0.667 P	0.433 N	2.797 P
L11	3.382 P	0.421 P	1.594 P	2.447 P
L12	2.149 P	0.915 P	0.064 N	1.344 E
L13	3.152 P	1.134 P	0.381 N	2.785 P
L14	3.271 P	1.521 P	2.316 P	3.400 P
L15	3.182 P	1.831 P	2.893 P	3.403 P
L16	2.411 P	0.134 N	0.125 N	3.443 P
L17	3.421 P	0.228 E	0.439 N	3.443 P
L18	2.576 P	0.237 E	0.324 N	3.421 P
LD neg	0.089 (0.07)	0.059 (0.056)	0.204 (0.129)	0.460 (0.259)

a L1 to L18, Borrelia-positive sera from MarDx; LD neg, Borrelia-negative sera.

b P, positive; N, negative; E, equivocal. For the LD-negative sera (*n* = 19, except for C6, for which *n* = 6), mean absorbance values (standard deviations) are shown.

**TABLE 2:** Comparisons of the DbpA/C6, DbpA, and OspC antigens for their ability to detect Borrelia-specific IgM antibodies in LD-positive sera

Serum^*a*^	Absorbance value and result^*b*^		
	DbpA/C6	DbpA	OspC
L1	0.020 N	0.005 N	0.720 P
L2	0.156 P	0.059 N	2.783 P
L3	0.215 P	0.053 N	3.219 P
L4	0.015 N	0.034 N	0.675 P
L5	0.020 N	0.000 N	1.126 P
L6	0.046 E	0.000 N	0.884 P
L7	0.042 N	0.036 N	0.614 P
L8	0.016 N	0.009 N	0.190 P
L9	0.019 N	0.011 N	0.840 P
L10	0.014 N	0.017 N	0.663 P
L11	0.021 N	0.007 N	1.493 P
L12	0.000 N	0.000 N	0.290 P
L13	0.085 P	0.006 N	1.307 P
L14	0.162 P	0.037 N	2.922 P
L15	0.287 P	0.402 P	3.234 P
L16	0.177 P	0.001 N	2.861 P
L17	0.151 P	0.013 N	3.461 P
L18	0.221 P	0.010 N	1.565 P
LD neg	0.008 (0.012)	0.015 (0.036)	0.049 (0.032)

a L1 to L18, LD-positive sera from MarDx; LD neg, LD-negative sera.

b P, positive; N, negative; E, equivocal. For the LD-negative sera (*n* = 19), mean absorbance values (standard deviations) are shown.

### Comparative testing of a panel of paired LD patient acute- and convalescent-phase sera from the CDC.

A panel of 15 pairs of acute- and convalescent-phase sera from physician-diagnosed early Lyme disease patients (9) was received from the CDC. The IgG and IgM status of each serum had already been determined at the CDC by the 2-tier method before their receipt. These sera were tested for IgG and IgM reactivity using the DbpA/C6 and OspC recombinant proteins, respectively. Cutoff and equivocal values for these tests were determined using a panel of 21 sera from negative-control subjects which had been accumulated in-house to date and which were included in each of the test runs. The individual results for both recombinant proteins as well as the 2-tier test are presented in Table 3 and summarized in Table 4. As shown, the DbpA/C6 and OspC antigens were successful in identifying a higher percentage of the sera, acute as well as convalescent phase, as being positive for LD-specific antibodies than did the 2-tier system (for a statistical evaluation, see Table S2 in the supplemental material). As shown, one pair of sera was completely negative by either test method.

**TABLE 3:** Comparison of DbpA/C6 (IgG) and OspC (IgM) with the 2-tier method for detecting LD-specific antibodies in a panel of paired acute- and convalescent-phase sera from the CDC

Serum pair	Phase	Absorbance value and/or result^*a*^						
		DbpA/C6 IgG	OspC IgM	Vidas^*b*^ IgG	Marblot^*c*^		2-tier	
					IgG	IgM	IgG	IgM
1	Acute	0.119 N	0.403 N	E	N	P	N	P
	Convalescent	2.989 P	1.268 P	P	N	P	N	P
2	Acute	3.219 P	0.955 P	P	N	P	N	P
	Convalescent	3.133 P	1.494 P	P	N	N	N	N
3	Acute	2.918 P	3.348 P	P	P	P	P	P
	Convalescent	3.169 P	2.742 P	P	P	P	P	P
4	Acute	3.280 P	1.347 P	P	N	P	N	P
	Convalescent	3.246 P	1.418 P	P	P	P	P	P
5	Acute	0.209 N	0.100 N	N	N	N	N	N
	Convalescent	0.221 N	0.106 N	N	N	N	N	N
6	Acute	3.075 P	3.422 P	P	N	P	N	P
	Convalescent	3.057 P	3.283 P	P	N	P	N	P
7	Acute	2.913 P	1.460 P	P	N	P	N	P
	Convalescent	3.046 P	0.918 P	P	N	P	N	P
8	Acute	3.325 P	0.219 N	P	P	N	P	N
	Convalescent	3.322 P	0.372 N	P	P	N	P	N
9	Acute	0.130 N	1.075 P	P	N	N	N	N
	Convalescent	0.000 N	0.628 E	E	N	N	N	N
10	Acute	1.554 P	0.410 N	P	N	N	N	N
	Convalescent	1.447 P	0.848 P	P	N	N	N	N
11	Acute	3.078 P	3.420 P	P	P	P	P	P
	Convalescent	3.074 P	3.185 P	P	P	P	P	P
12	Acute	3.094 P	1.965 P	P	P	N	P	N
	Convalescent	2.503 P	2.399 P	P	P	N	P	N
13	Acute	1.370 P	1.571 P	P	N	N	N	N
	Convalescent	3.302 P	3.167 P	P	P	N	P	N
14	Acute	1.702 P	0.669 E	P	P	N	P	N
	Convalescent	3.074 P	0.728 E	P	P	P	P	P
15	Acute	0.861 P	3.113 P	P	N	P	N	P
	Convalescent	2.187 P	2.907 P	P	N	P	N	P
LD negative		0.111 (0.139)	0.154 (0.149)					

a P, positive; N, negative; E, equivocal. For the LD-negative sera (*n* = 21), mean absorbance values (standard deviations) are shown.

b First tier of the 2-tier method.

c Second tier of the 2-tier method.

**TABLE 4:** Summary of the comparative results with the panel of acute/convalescent-phase sera pairs from Table 3

Sera	Antibody	% LD positive (no. positive/total)	
		DbpA/C6-OspC^*a*^	2-tier method
Acute phase	IgG	80 (12/15)^*b*^	33 (5/15)
	IgM	67 (10/15)^*c*^	53 (8/15)
	IgG/IgM	87 (13/15)^*c*^	73 (11/15)
Convalescent phase	IgG	87 (13/15)^*b*^	47 (7/15)
	IgM	73 (11/15)^*c*^	53 (8/15)
	IgG/IgM	87 (13/15)^*c*^	73 (11/15)

a DbpA/C6 for detecting IgG and OspC for detecting IgM.

b Statistically superior (see Table S2 in the supplemental material).

c No statistical difference (see Table S2 in the supplemental material).

### Comparative evaluation of a 279-serum LD premarketing panel from the CDC.

To test the DbpA/C6 and OspC recombinant antigens in a larger blind study, the CDC generously provided a panel of 279 sera derived from LD patients and various negative-control subjects (9). Each of these sera was tested by ELISA for IgG antibodies to DbpA/C6 and for IgM antibodies to OspC. Included in each test run were 24 “in-house” as well as MarDx negative-control sera from individuals without LD. After the ELISA results were obtained and recorded, the CDC provided a table with the identification of each serum as well as the individual IgG and IgM test results, which they had previously obtained by the 2-tier method. Eighty-nine of these sera were from physician-diagnosed LD-positive patients. The remaining 190 sera were from patients with no history of LD (9). These included 100 sera from healthy individuals (50 from areas where LD is not endemic and 50 from areas where it is endemic). It also included 90 sera from patients with diseases that can mimic some symptoms of LD and/or induce cross-reactive antibodies to Borrelia antigens, thus complicating an accurate diagnosis. These included 15 sera each from patients with fibromyalgia, mononucleosis, multiple sclerosis, periodontitis, rheumatoid arthritis, and syphilis.

**FIG 2:**
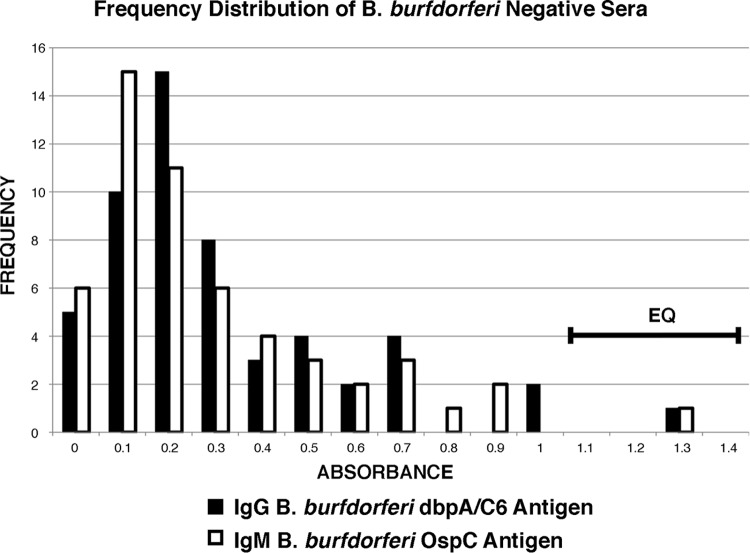
Frequency distribution graph showing the relation of the cutoff and equivocal values to the 54 negative sera used to generate them. These values were used to declare the positive and negative status of each serum in the comparative tests of the 279-serum premarketing panel from the CDC. The cutoff value was 1.1, and the equivocal range was between 1.1 and 1.4 (EQ).

To determine cutoff and equivocal values for this 279-serum panel, the results of 54 sera from subjects without LD were used. These included the 24 “in-house” and MarDx negative-control sera, included in each test run, in combination with the results for 30 randomly selected (using a program from random.org) sera from the 100 healthy individuals from areas where LD is endemic or not endemic. The inclusion of the 30 randomly selected results was an attempt to increase the accuracy of the cutoff and equivocal values by increasing the number and diversity of the negative sera used to generate them. The results for the 30 randomly selected sera were then omitted from any of the downstream comparison calculations (leaving 160 of the original 190 negative sera for the comparisons). To show how these 54 LD-negative sera were distributed relative to the calculated cutoff and equivocal range values, a frequency distribution graph was created for both IgG and IgM. The cutoff value was set at 3 standard deviations (SDs), and the equivocal range was set between 3 and 4 SDs from the mean (Fig. 2). This gave a calculated cutoff value for both IgG and IgM of 1.1 and an equivocal range of between 1.1 and 1.4 (Fig. 2, EQ). As can be seen, these values occur at the extreme ends of the tails of each curve and as such were positioned to minimize the number of false-positive determinations (see Discussion).

The complete test results for the 279-serum premarketing panel, comparing the recombinant antigens with the 2-tier method, are shown in the Table S1 in the supplemental material. This table allows individual comparisons of each of the sera tested. Shown are the absorbance values for each serum obtained by ELISA, using as antigens DbpA/C6 for the detection of IgG and OspC for the detection of IgM, along with their declared IgG and IgM status, based on the calculated cutoff and equivocal range values. These results are then compared with the individual test results from each tier of the 2-tier method conducted at the CDC, including the identity of the positively scored IgG or IgM bands from each WB. For comparison purposes, it is noted that the molecular masses of the native DbpA and OspC proteins are considered to be 18 and 23 kDa, respectively (10, 11).

Table 5 summarizes the results for all 249 sera (omitting the results for the 30 sera used in the calculations of the cutoff and equivocal values) from the 279-serum premarketing panel with regard to their declared clinical status as being from subjects with or without LD. The comparisons are grouped into stage I LD, stage II and III LD, and non-LD categories. The 2-tier method uses the combined results of both the IgG and IgM WB determinations to declare an overall positive or negative status for each serum. When the results using the DbpA/C6 (IgG) and OspC (IgM) antigens are combined, they are statistically the same as those from the 2-tier method for identifying LD-negative subjects (99% versus 100%) as well as stage II and III LD patient samples (100% versus 100%). However, the combined results of both the DbpA/C6 and OspC tests were statistically superior (see Table S2 in the supplemental material) to those of the 2-tier method in identifying stage I LD patients (80% versus 63%). The data also show that even when considering the DbpA/C6 IgG determinations alone, the results were still equivalent to those of the 2-tier test in identifying LD negative-control sera (100% versus 100%) and in identifying stage II and III LD patient sera (97% versus 100%) but again were statistically better than those of the 2-tier test in identifying stage I LD patient sera (78% versus 63%).

**TABLE 5:** Summary of the comparative results with the 279-serum premarketing panel from the CDC

Sera	Antibody	% LD positive (no. positive/total)	
		DbpA/C6-OspC^*a*^	2-tier method
Stage I LD	IgG	78 (46/59)^*b*^	29 (17/59)
	IgM	64 (38/59)^*c*^	51 (30/59)
	IgG/IgM	80 (47/59)^*a*^	63 (37/59)
Stage II and III LD	IgG	97 (29/30)^*c*^	87 (26/30)
	IgM	43 (13/30)^*c*^	53 (16/30)
	IgG/IgM	100 (30/30)^*c*^	100 (30/30)
LD-negative subjects	IgG	0 (0/160)^*c*^	0 (0/160)
	IgM	1 (2/160)^*c*^	0 (0/160)

a DbpA/C6 for detecting IgG and OspC for detecting IgM.

b Statistically superior (see Table S2 in the supplemental material).

c No statistical difference (see Table S2 in the supplemental material).

## DISCUSSION

As previously indicated, at the beginning of the study more than a dozen recombinant B. burgdorferi genes, whose encoded proteins were considered to be important in the serology of LD, were cloned, expressed in E. coli, and purified. The purpose for making these recombinant proteins was to identify candidate antigens which could be used to develop a simplified assay for the serological confirmation and diagnosis of LD. Each protein, therefore, was carefully screened for its ability to react with IgG or IgM antibodies in human sera which had previously been determined by WB to contain both types of anti-Borrelia antibodies. Most of these antigens were quickly eliminated from further study because of poor to moderate reactivity with the positive-control sera. Three of the recombinant proteins (p39, p41, and VlsE), which were highly reactive with both IgG and IgM antibodies in LD-positive sera, also showed a high degree of reactivity with certain of the negative-control subject samples. In order to justify eliminating these antigens as possible candidates for continued study, further investigation of their reactivity with LD-negative subject sera was warranted. It seemed conceivable that some of the observed reactions with the sera from LD-negative subjects might simply be a result of reactions with E. coli proteins that may have copurified with the recombinant antigens. This possibility was tested in two ways: (i) the LD negative-control sera were absorbed with E. coli lysates prior to the performance of ELISAs using VlsE, p39, or p41 as the antigen, and (ii) p41was further purified, to >99%, using isoelectric focusing (IEF). However, neither the absorption studies nor the further purification of p41 resulted in any observable difference in the noted reactivity with the LD negative-control sera, indicating that it was not E. coli proteins that were involved in these reactions. A more likely explanation was that these reactions were due to cross-reactive antibodies present in these particular negative-control subjects, possibly generated by exposure to other agents possessing proteins with epitopes that were structurally similar to those found in VlsE, p39, or p41. The ability of an antigen to react with cross-reacting antibodies is a difficult challenge to overcome in trying to develop a simplified serological test, especially one which emphasizes specificity. Therefore, these antigens were also eliminated from further testing. In the evaluation process, two antigens emerged, DbpA and OspC, which were highly reactive with IgG or IgM antibodies in LD-positive patient sera, respectively, and which were nonreactive with any of the LD negative-control sera.

It had been reported (12) that the synthetic peptide C6 was both a sensitive and specific marker for detecting anti-Borrelia IgG antibodies in LD patient sera. It seemed reasonable to assume, therefore, that if DbpA was fused with the small C6 peptide, it could result in a chimeric protein with an enhanced ability to detect IgG anti-Borrelia antibodies in LD patient sera. The comparative studies described above showed this to be true. Therefore, DbpA/C6 was used in all testing of the LD serum panels received from the CDC.

As previously noted, recombinant OspC had emerged as being a very good antigen for the detection of IgM anti-Borrelia antibodies. However, it was expressed in E. coli in small amounts, which greatly hindered its purification. In an effort to increase expression of OspC, it was fused with a portion of the E. coli maltose binding protein (MBP) gene. This generated a fusion protein with expression levels greater than 5-fold over those of unfused OspC, which markedly facilitated its purification. ELISA testing of this MBP/OspC fusion protein showed a small overall increase in its reactivity with Borrelia IgM-positive patient sera compared to that of OspC alone, probably reflecting a higher specific activity due to enhanced purification. However, importantly, it also indicated that the fusion with this piece of MBP did not alter the folding of OspC in a manner that observably hindered antibody reactions with specific epitopes. Another important observation using this version of OspC was that no increase in reactions with LD-negative subject sera was noted. This fusion protein, designated simply OspC, was used in all the tests reported in this paper.

The receipt of well-characterized panels of LD-positive and -negative sera from the CDC allowed the opportunity to test the two antigens, DbpA/C6 and OspC, much more extensively. The first of such tests reported here was those of physician-diagnosed LD patient acute- and convalescent-phase serum pairs. For these tests, the only negative sera that could be included for the establishment of cutoff and equivocal values were those which had been accumulated “in-house” to that date and which had been given to us by MarDx. Although specificity could not be determined, the sensitivity of the ELISA reactions using the two recombinant proteins, DbpA/C6 for IgG and OspC for IgM, exceeded those of the 2-tier test for both acute- and convalescent-phase sera.

The final panel received from the CDC contained 279 sera. Each was labeled with only a number. After all the tests had been run and the data obtained and recorded, the CDC supplied the key to the panel. The cutoff and equivocal values (calculated from a pool of negative sera as described in Results) were set at 3 and 4 standard deviations from the mean, respectively, to emulate the 2-tier test method in trying to maximize specificity without excessively compromising sensitivity (4). The practical reason for this was that with millions of LD tests conducted each year, even a 1% false-positive rate could result in tens of thousands of people annually being unnecessarily treated for LD (5, 8). Using the established cutoff and equivocal range values to predict a positive or negative status for each serum, a direct comparison of the ELISA using the two recombinant antigens (DbpA/C6 and OspC) with the 2-tier test method was made. Besides the DbpA/C6-OspC ELISA being simpler to perform and to interpret, the major notable difference between it and the 2-tier test method was its greater capacity to identify stage I LD patient sera compared to that of the 2-tier method. This is significant, because early confirmation of LD in a patient can stimulate the initiation of specific treatment for the disease, thereby lowering the patient's potential risk for developing the debilitating complications associated with the later stages of illness. With regard to early detection of LD, Table S1 in the supplemental material shows that the Vidas IgG ELISA, used as a screening test in the 2-tier method, compared equivalently to the DbpA/C6-OspC ELISA in identifying stage I LD patient sera. However, a significant disadvantage to the Vidas IgG test is that it also gave LD-positive status to 53% (8/15) of the subjects in the mononucleosis group and 87% (13/15) of the subjects in the syphilis group, compared to 7% (1/15) and 0% (0/15), respectively, for the DbpA/C6-OspC test. The WB assays in the 2-tier test identified all the sera from the subjects in these two groups as anti-Borrelia antibody negative.

In summary, this study has shown that the two recombinant proteins described here (DbpA/C6 and OspC) are excellent antigen candidates for the establishment of a simple and reliable 1-tier ELISA for the serological confirmation and diagnosis of LD. Using these antigens, such a test could have the specificity of the second-tier WB assays of the 2-tier test method but the sensitivity of the first-tier screening assay. As such, it could possess a greater capacity to identify Borrelia-specific antibodies in the sera of patients with stage I LD and still be highly discriminatory toward the sera of LD-negative subjects. In addition, such a test would be faster and easier to perform, simpler to interpret, and less expensive than the 2-tier test method.

## MATERIALS AND METHODS

### Bacteria, plasmid, C6 peptide, and sera.

Borrelia burgdorferi strain B31 cells were a gift from MarDx Diagnostic, Inc., Carlsbad, CA. E. coli BL21(DE3)/pLysS One Shot and Top 10 chemically competent cells were purchased from Invitrogen Life Technologies, Carlsbad, CA.

An expression vector containing an ampicillin resistance gene, a T7 promoter, and code for six histidines upstream of a multiple-cloning site was used for all protein expressions. The C6 peptide (CMKKDDQIAAAMVLRGMAKDGQFALK), whose amino acid structure was deduced from the invariable region 6 (IR6) of the *vlsE* gene (12), was produced by Biomatik, Cambridge, Ontario, Canada.

For the initial testing of the developed antigens, control sera from LD-positive patients as well as LD-negative subjects were generously supplied by MarDx Diagnostics, Inc. (Carlsbad, CA). These control sera had been determined by MarDx to be LD IgG and IgM positive or LD negative using their Marblot WB assays. In addition, sera from healthy subjects with no known history of LD were obtained locally and designated “in-house” negative-control sera. These were confirmed negative by WB testing or by ELISA using B. burgdorferi B31 lysates as the antigen (not shown). Well-characterized test panels of LD-negative subject sera and LD-positive patient sera were kindly provided by the CDC (Fort Collins, CO). The acquisition and acceptance of these sera by the CDC for use in Lyme disease diagnostic test development and evaluation were as described previously (9). For this study, the LD panels from the CDC were supplied in numbered tubes only. The final key was not provided until after the test results were recorded.

### Cloning of B. burgdorferi genes.

DNA and RNA were extracted from pelleted B. burgdorferi cells using the DNeasy and RNeasy kits, respectively, from Qiagen Sciences, Inc., Germantown, MD, USA, according to the manufacturer's instructions. Primers for the PCRs (Table 6) were purchased from the University of Utah Cores Labs, Salt Lake City, UT. PCRs were carried out using the SuperScript III one-step reverse transcription-PCR (RT-PCR) or the Platinum *Taq* DNA polymerase high-fidelity kit from Invitrogen Life Technologies.

**TABLE 6:** Primers used in PCR amplification of specific B. burgdorferi and E. coli genes

Gene	Sequence^*a*^	
	5′ oligonucleotide primer	3′ oligonucleotide primer
*dbpA*^*b*^	GGATCCCTCGAGATGATTAAATGTAATAATAAA	AAGCTTCTGCAGGTTATTTTTGCATTTTTCATC
*ospC*^*b*^	GGATCCCTCGAGATGAAAAAGAATACATTAAGT	AAGCTTCTGCAGAGGTTTTTTTGGACTTTCTGC
*vlsE*^*c*^	CTCGAGCTGCAGATGAAGAAGGATGATCAGATT	GAATTCGGATCCACCATCCTTCACAGCAAACTT
MBP gene^*d*^	CTGCAGCTCGAGATGAAAATCGAAGAAGGTAAA	GAATTCCTGCAGCAGATCTTTGTTATAAATCTG

a Underlined regions represent the gene-specific sequences.

b B. burgdorferi gene.

c IR6 region of the B. burgdorferi *vlsE* gene.

d MBP, E. coli maltose binding protein.

PCR amplification of the *dbpA*, *ospC*, and *vlsE* IR6 genes from B. burgdorferi as well as the E. coli maltose binding protein (MBP) gene (representing amino acids 1 to 122 of MBP) yielded PCR products with approximate sizes, in an agarose gel, corresponding to the expected sizes of 597, 653, 105, and 390 bp (including primer arms), respectively (not shown). These amplicons were cut with the appropriate restriction enzymes and cloned into the multiple-cloning site of the expression plasmid described above in frame with the code for six histidines. The *dbpA* gene was cloned by itself as well as in tandem with the *vlsE* IR6 gene (with *vlsE* IR6 immediately behind *dbpA*). The *ospC* and MBP genes were also cloned in tandem, with the MBP gene directly in front of *ospC*. The expression plasmids containing the *dbpA*, *dbpA*/IR6, and *ospC* genes yielded recombinant proteins DbpA, DbpA/C6, and OspC, with approximate molecular masses of 19, 27, and 37 kDa, respectively (not shown).

### Recombinant protein expression and purification.

Expression plasmid DNA was purified using the QIAfilter plasmid midikit from Qiagen (Valencia, CA), transformed into chemically competent BL21(DE3)/pLysS cells as directed by the manufacturer, and grown overnight on LB agar plates containing 100 μg/ml of ampicillin. Transformed colonies were harvested into LB medium and used to inoculate 3 × 2 liters of LB medium containing ampicillin in 3-liter baffled flasks. The flasks were rotated at 120 rpm for 4 to 5 h at 37°C until the turbidity reached an optical density at 600 nm (OD_600_) of approximately 0.8. Recombinant protein expression was induced with the addition of IPTG (isopropyl-β-d-thiogalactopyranoside) to 1 mM and the flasks incubated with shaking for 2 h. Cells were concentrated by centrifugation, washed once in phosphate-buffered saline (PBS), suspended in 35 ml of binding buffer (20 mM sodium phosphate, 500 mM sodium chloride, pH 7.8) containing 7 M guanidine-HCl, and set at −80°C. The suspension was thawed, sonicated (3 20-s pulses using a Sonic Dismembrator 550 at a setting of 8), and centrifuged at 32,000 × *g* for 30 min. The His-tagged proteins were then isolated using Ni-nitrilotriacetic acid (NTA)–agarose (Qiagen Inc., Valencia, CA) as directed by the manufacturer. The isolated proteins were dialyzed (12,000- to 14,000-molecular-weight-cutoff dialysis tubing) overnight against 1 liter of binding buffer containing 2 M urea. The protein concentration was determined, and the protein was diluted to 1 mg/ml in binding buffer containing 2 M urea and stored frozen at −80°C.

### Protein determination and SDS-PAGE.

Total protein was determined by the Bradford method (13) using dye and bovine gamma globulin standards from Bio-Rad Laboratories, Hercules, CA. SDS-PAGE analysis was carried out using Invitrogen Life Technologies NuPAGE 4 to 12% Bis-Tris precast polyacrylamide gels and run on the Invitrogen Novex Mini-Cell electrophoresis apparatus. Separated proteins were stained with 0.25% Brilliant Blue R (Sigma Chemical Co., St. Louis, MO).

### ELISA reactions.

Recombinant antigens were diluted to 10 μg/ml in glycine buffer (0.05 M Glycine [pH 9], 137 mM NaCl), 0.1 ml/well was added to Nunc F8 Maxisorb Immuno Module ELISA strips, and the strips were incubated overnight at 4°C. The wells were washed 3 times with 0.3 ml/well of TBS (0.05 M Tris [pH 7.4], 137 mM NaCl) containing 0.05% Tween 20, and then 0.2 ml/well of blocking buffer was added and the wells incubated at room temperature for 40 min. For IgG detection, the blocking buffer was TBS plus 5% milk diluent/blocking solution from Kirkegaard and Perry Laboratories, Inc., Gaithersburg, MD. For IgM detection, the blocking buffer was TBS plus 10% normal goat serum (HyClone, Logan, UT). Sera were diluted 100-fold for IgG assays and 50-fold for IgM assays in their respective blocking buffers. The secondary antibody used in the ELISA reactions was horseradish peroxidase (HRP)-conjugated goat anti-human IgG or IgM (American Qualex, San Clemente, CA) diluted 1,000-fold in blocking buffer. The substrate was *o*-phenylenediamine dihydrochloride (Sigma Chemical Co., St. Louis, MO) used at 3 mg/ml in citrate buffer (0.024 M citric acid, 50 mM Na_2_HPO_4_, pH 5). For all assays, blank wells were prepared exactly as they were for the test wells except that no antigen was present in the coating buffer. At the conclusion of the assay, the absorbance value from each blank well was subtracted from that of their corresponding test well, yielding a net absorbance value for each individual serum tested.

## Supplementary Material

Supplemental material
