# Supplementary material for: Improved Serodiagnostic Performance for Lyme Disease by Use of Two Recombinant Proteins in Enzyme-Linked Immunosorbent Assay Compared to Standardized Two-Tier Testing
Source: J Clin Microbiol. 2017 Sep 25;55(10):3046–56. doi: 10.1128/JCM.01004-17 (PMC5625391; doi:10.1128/JCM.01004-17)
Supplement: Supplemental material [file supp_55_10_3046__index.html]

Supplemental material 

# Improved Serodiagnostic Performance for Lyme Disease by Use of Two Recombinant Proteins in Enzyme-Linked Immunosorbent Assay Compared to Standardized Two-Tier Testing

## Supplemental material

- Supplemental file 1 -

  Table S1 (Results of comparison of dbpA/C6 and OspC with the 2-tiered method using the CDC 280 serum premarketing test panel)

  XLSX, 38K
- Supplemental file 2 -

  Table S2 (Noninferiority, superiority, and equivalence testing of Lyme data)

  PDF, 187K
