# Supplementary material for: Improved Serodiagnostic Performance for Lyme Disease by Use of Two Recombinant Proteins in Enzyme-Linked Immunosorbent Assay Compared to Standardized Two-Tier Testing
Source: J Clin Microbiol. 2017 Sep 25;55(10):3046–56. doi: 10.1128/JCM.01004-17 (PMC5625391; doi:10.1128/JCM.01004-17)
Supplement: Supplemental material [file JCM.01004-17_zjm999095665s2.pdf]

Table S2. Noninferiority, Superiority and Equivalence Testing of Lyme Data
22 June 2017, Dr. H. Dennis Tolley

The purpose of this note is to report the results of statistical comparisons of the RSL diagnostic procedures relative to the CDC procedures. All tests done with type I error probability (alpha) of 0.05.

Table A

Comparison	RSL	2-Tier	Result
Table 4 Line 1	12/15	5/15	Superiority test, Reject H_o conclude >5% superior.
Table 4 Line 2	10/15	8/15	No Statistical Difference.
Table 4 Line 3	13/15	11/15	No Statistical Difference.
Table 4 Line 4	13/15	7/15	Superiority test, Reject, Conclude >5% superior.
Table 4 Line 5	11/15	8/15	No Statistical Difference.
Table 4 Line 6	13/15	11/15	No Statistical Difference.
Table 5 Line 1	46/59	17/59	Superiority test, Reject, Conclude >10% superior.
Table 5 Line 2	38/59	30/59	No Statistical Difference.
Table 5 Line 3	47/59	37/59	Superiority test, Reject, Conclude >1% superior.
Table 5 Line 4	29/30	26/30	No Statistical Difference.
Table 5 Line 5	13/30	16/30	Fail to reject, cannot conclude noninferiority at 1%.
Table 5 Line 6	30/30	30/30	No Statistical Difference.
Table 5 Line 7	160/160	160/160	No Statistical Difference.
Table 5 Line 8	158/160	160/160	Conclude equivalent at 5% but fail to reject at 1%.

Four different hypothesis tests were done: a statistical difference (the classic hypothesis), superiority hypothesis, the inferiority hypothesis and the equivalence hypothesis. The decision

as to which of these were done was based on the observed counts. All tests were performed using the Exact test procedure and a bootstrap procedure. Statistical analyses were done using NCSS (NCSS 11 Statistical Software (2016), NCSS, LLC, Kaysville, Utah, ncss.com/software/ncss).

Neither of the hypotheses of noninferiority and equivalence for Table 5 line 5 could be rejected and consequently we could not conclude that the RSL test was not inferior at the 1% difference nor was it conclusively equivalent. The major reason for this was the low number of observations.

The equivalence hypothesis for Table 5 Line 8 was rejected for a difference of 5% but not for a difference of 1%. Consequently, we can conclude that if a significant difference in probability of 5 percentage points is tested, the dbpA/C6-OspC procedure is equivalent to the Two Tier. However, if the posited difference is only 1 percentage point, there is insufficient information to conclude the two procedures are different.

Table 5 Line 1 concludes RSL is superior even if one requires a 10 percentage point difference for superiority. Table 4 Line 4 concludes dbpA/C6-OspC is superior even if one requires a 5 percentage point difference for superiority.
